# Breastfeeding support for adolescent mothers: similarities and differences in the approach of midwives and qualified breastfeeding supporters

**DOI:** 10.1186/1746-4358-1-23

**Published:** 2006-11-25

**Authors:** Victoria Hall Moran, Fiona Dykes, Susan Burt, Christina Shuck

**Affiliations:** 1 Maternal and Infant Nutrition and Nurture Unit (MAINN), Faculty of Health, University of Central Lancashire, Preston, UK; 2 Sharoe Green Unit, Royal Preston Hospital, Preston, Lancashire, UK

## Abstract

**Background:**

The protection, promotion and support of breastfeeding are now major public health priorities. It is well established that skilled support, voluntary or professional, proactively offered to women who want to breastfeed, can increase the initiation and/or duration of breastfeeding. Low levels of breastfeeding uptake and continuation amongst adolescent mothers in industrialised countries suggest that this is a group that is in particular need of breastfeeding support. Using qualitative methods, the present study aimed to investigate the similarities and differences in the approaches of midwives and qualified breastfeeding supporters (the Breastfeeding Network (B*f*N)) in supporting breastfeeding adolescent mothers.

**Methods:**

The study was conducted in the North West of England between September 2001 and October 2002. The supportive approaches of 12 midwives and 12 B*f*N supporters were evaluated using vignettes, short descriptions of an event designed to obtain specific information from participants about their knowledge, perceptions and attitudes to a particular situation. Responses to vignettes were analysed using thematic networks analysis, involving the extraction of basic themes by analysing each script line by line. The basic themes were then grouped to form organising themes and finally central global themes. Discussion and consensus was reached related to the systematic development of the three levels of theme.

**Results:**

Five components of support were identified: emotional, esteem, instrumental, informational and network support. Whilst the supportive approaches of both groups incorporated elements of each of the five components of support, B*f*N supporters placed greater emphasis upon providing emotional and esteem support and highlighted the need to elicit the mothers' existing knowledge, checking understanding through use of open questions and utilising more tentative language. Midwives were more directive and gave more examples of closed questions. These differences could reflect the considerable emphasis upon person-centred approaches within the B*f*N curriculum and, in the case of midwives, the bureaucratic and institutional constraints upon them making it difficult, if not impossible, to take time and touch base with women.

**Conclusion:**

Follow up ethnographic work is required to assess the differences in the supportive approaches of B*f*N supporters and midwives in the practice areas. Such research, which specifically focuses upon how the different approaches are received and experienced by parents, is required before meaningful policy and practice recommendations can be made.

## Background

The protection, promotion and support of breastfeeding are now major public health priorities, as emphasised in the *Global Strategy for Infant and Young Child Feeding *[1]. The Global Strategy aims to "improve – through optimal feeding – the nutritional status, growth and development, health, and thus the survival of infants and young children" [1](see page 6]. Central to this is the recommendation that infants should be exclusively breastfed for the first six months of life and thereafter receive nutritionally adequate and safe complementary foods with breastfeeding continuing for up to two years of age or beyond. Within the Global Strategy the statement is made that:

"Mothers should have access to skilled support to help them initiate and sustain appropriate feeding practices, and to prevent difficulties and overcome them when they occur. Knowledgeable health workers are well placed to provide this support, which should be a routine part not only of regular prenatal, delivery and postnatal care but also of services provided for the well baby and sick child" [1](see page 12].

There has been a wealth of research conducted amongst diverse populations around the world, which has attempted to define the factors influencing the initiation and duration of breastfeeding [2-7]. It is now well established that skilled support, voluntary or professional, proactively offered to women who want to breastfeed can increase the initiation and/or duration of breastfeeding [2,3,5-7]. It is also clear that a major reason for early cessation of breastfeeding relates to women's perceived difficulty with breastfeeding [7-9]. It is axiomatic, therefore, that an absence of skilled support is a key factor in shortening the duration of breastfeeding. Specific groups, such as families from socially deprived settings and adolescent parents, have particularly low levels of breastfeeding uptake and continuation in industrialised countries [10,11], suggesting that such groups may be in particular need of breastfeeding support. The provision of skilled support to such groups is one way of reducing the cycle of nutritional deprivation between mother and child [12].

Breastfeeding women are carrying out a partially learned activity. When breastfeeding is not the cultural norm, there are low levels of knowledge and support within a given community and therefore health professionals become a key source of necessary support [7,13] and [14]. However, although health professionals can positively influence breastfeeding women through providing effective support, they can also be a negative source of support when they provide women with inconsistent, inaccurate and/or inadequate breastfeeding information and recommendations [13,15-19].

It has been suggested that, in view of the public health importance of breastfeeding, there is a need to develop and evaluate supplementary support strategies as part of routine health service provision [6,7]. A number of peer support programmes have been established and evaluated [9,10,20-24]. Drawing study findings together, two systematic reviews concluded that peer support can be effective in supporting women to continue breastfeeding [6,7]. However, findings from the reviews reflect a lack of clarity regarding the types of peer support being researched. In the UK, for example, there are two types of non-health professional supporter. There are accredited breastfeeding supporters/counsellors working within voluntary organisations who receive 1–2 years of intensive training and there are peer supporters who commonly receive approximately 20 hours of training.

The Breastfeeding Network (B*f*N) is a voluntary organisation that provides support to breastfeeding women. Although UK based, the training and person-centred approaches utilised by the B*f*N are similar to those used by international organisations such as La Leche League (LLL) and the Association for Breastfeeding Mothers (ABM). This paper therefore presents a case study of voluntary support organisations which has international applicability.

The breastfeeding training of B*f*N supporters is underpinned by Rogers' theory of client- or person-centred counselling [25]. The person-centred approach places high value on the experience of the individual and the importance of her/his subjective reality [26]. The counsellor's role is not to guide or direct but to create an environment within which the individual can discover and develop her own inner resources to deal with challenging situations. The creation of this environment requires the counsellor to be genuine or congruent, to offer unconditional positive regard and acceptance, and to feel and communicate a deep empathic understanding. These three elements are often referred to as the core conditions of the person-centred approach.

To enable their supporters to achieve these conditions in their relationships with breastfeeding women the B*f*N training programme aims to increase self-awareness, provide opportunities for reflection on personal experiences and attitudes and develop listening and communication skills. Trainees learn how to work with women both face-to-face and on the telephone and undertake a series of assignments, including a taped role-play that assesses their use of listening and counselling skills. For a year following qualification the probationary supporters receive formal supervision every six weeks from their tutor and informal guidance as required. In addition, all B*f*N registered breastfeeding supporters undergo ongoing training and regular supervision and are also required to have had the personal experience of breastfeeding their own child. The B*f*N is a member of the British Association for Counselling and Psychotherapy, committing supporters to following the Ethical Framework for Good Practice in Counselling and Psychotherapy.

In contrast, whilst UK midwifery training may extend over 18 months for qualified nurses and four years for direct entrants, a relatively small proportion of this time is devoted to breastfeeding education. The content of this education may vary between educational establishments but generally it conforms to the traditional biomedical model of health professional training with little or no emphasis on counselling skills. It is not unreasonable to assume, therefore, that the nature of the breastfeeding support provided by health professionals may differ to that provided by voluntary breastfeeding supporters.

Previous quantitative research has revealed that breastfeeding support skills scores, evaluated using a pre-validated measurement tool, were significantly higher in B*f*N supporters than midwives, many of whom had undergone UNICEF UK BFI training [27]. The need for additional qualitative research that explores the different elements of breastfeeding support strategies and the mechanisms by which they operate has been emphasised [6]. Using qualitative methods, the present study aimed to investigate the similarities and differences in the approaches of midwives and qualified breastfeeding supporters (B*f*N supporters) in supporting breastfeeding adolescent mothers.

## Methods

The study was conducted in the North West of England between September 2001 and October 2002.

### Measurement of supportive approaches

The supportive approaches of midwives and B*f*N supporters with regards to breastfeeding were explored qualitatively using vignettes. As this is an under-explored area needing preliminary work before hypotheses could be tested, a qualitative study was considered appropriate in order to support understanding. Vignettes are short descriptions of an event, which may be fact or fiction, and are designed to obtain specific information from participants about their knowledge, perceptions and attitudes to a particular situation. They have been used both within quantitative and qualitative research, and are considered particularly effective tools for examining different groups' interpretation of a uniform situation [28]. Used within the qualitative paradigm, vignettes are useful when exploring the complexities of the different responses [29]. One of the acknowledged difficulties associated with the use of vignettes is the extent to which the response to the vignette reflects the action of the practitioner in the 'real' situation [30]. However, in this study it was the knowledge and attitudes of the two groups that were of interest and not their translation into action as this can be affected by a diversity of uncontrollable variables including, for example, time constraints.

### Development of the vignettes

The first stage of vignette development involved eliciting the experiences of breastfeeding mothers using focus groups. Focus groups were felt to be useful for this purpose because focus groups facilitate both the exploration of potentially embarrassing issues and the establishment of group norms [31,32]. The transcribed data were analysed using thematic networks analysis (as described later). Analysis of the focus groups yielded five themes: feeling watched and judged, lacking confidence, tiredness, discomfort and sharing accountability. The content of these themes have been discussed in greater detail in an earlier paper [11]. Four vignettes were developed from the themes that arose from this analysis (Table 1). The vignettes were mailed to the respondents together with written instructions on how to complete the task. For each vignette, respondents were asked to comment using free text on the scenario and describe what they might do or say.

**Table 1:** Details of the vignettes

*Vignette 1* Estelle, aged 16, has a baby aged seven days old. She tells you how she felt in hospital. Now that she is home she still feels the same: "*I don't feel confident...What if I'm not doing it *(breastfeeding) *right, am I going to make her poorly? I'm worrying about things like that.*"

*Vignette 2* Chantelle, aged 17, approaches you and expresses anxiety about her milk supply. Her baby is 3 weeks old. This followed a visit to the baby clinic last week when the following conversation took place: "*I wasn't asked how I was feeding. I was asked *'*How many ounces is he having?*'* And I said I breastfeed, but she just asked again *'*How many ounces?*'"

*Vignette 3* Jasmine, aged 14, has a baby, Ben, aged nearly 4 weeks old. She comes to you and says that she is thinking about changing to bottle feeding. She says: "*I think that you always feel that you're being watched to see whether you're able to look after your baby. It puts you in a position of being so nervous about whether you're doing it right 'cos the older people are looking at what you're doing. They don't expect you to be able to do it because you're so young.*"She tells you that she felt this way in hospital and now she feels the same way about feeding out in public.

*Vignette 4* Sally, who has a baby, Tyler, aged two weeks old, tells you that she is feeling very tired but she has a very supportive partner who has helped her. She said: "*He was so pleased and proud that he could give her a bottle feed of SMA when he came home from work, before she went to bed. I could actually sit there and she was getting fed and I could have a break as well. Strange isn't it? I don't feel so tied down that way but I still want to breastfeed.*"

### Sample recruitment and selection

Ethics approval for the study was granted by the Local Research Ethics Committee and relevant University Ethics and scientific review committees. Standard ethical procedures to obtain informed written consent, to protect participants' autonomy and to ensure anonymity and confidentiality of data were employed.

Focus group participants were adolescent mothers, who were recruited by the Teenage Pregnancy Coordinator, a midwife employed by the maternity unit to provide support for all adolescent mothers through pregnancy and following birth. Inclusion criteria were that the participants were aged 13–19 years, with a term healthy infant, and must have breastfed at least once. Adolescents unable to communicate in English, with known learning difficulties or mental health problems were excluded, as were those with a baby who was unwell and/or admitted to the neonatal unit. An adolescent cohort was selected as this group appears to be one that is in particular need of effective breastfeeding support.

Midwife participants were employed by a single NHS Trust. Permission to access the midwives and to use the hospital setting was obtained from the Head of Midwifery at the Trust. Midwives working as core staff on delivery ward or as core staff on antenatal ward were excluded from the study due to their limited contact with postnatal women. Fifty midwives were selected at random, using a random number table, and invited to participate in the study. Twelve midwives agreed to participate.

B*f*N supporters were contacted through a B*f*N trainer. The limited number of supporters in the region made random sampling impossible and thus a convenience sample of 18 supporters was invited to participate. Twelve supporters responded to the invitation.

### Procedure

#### Focus groups

Two focus groups were conducted in a quiet room at the local hospital. Two researchers conducted the focus groups, facilitating methodological rigour [33]. One researcher facilitated the discussion while another made comprehensive field notes relating to group dynamics and non-verbal behaviour. The focus groups were taped and transcribed. Following the first focus group, two of the participants were invited to join the project steering committee to help plan, develop and evaluate the ongoing research. The focus group data were utilised to develop four vignettes (as described below).

#### Vignettes

Midwives and B*f*N supporters were mailed pre-coded vignettes together with written instructions on how to complete the task. A reply paid envelope was provided and their anonymised responses were requested within two weeks.

### Analysis

The transcribed data from the focus groups and vignettes were analysed separately using thematic networks analysis [34]. This involved extracting basic themes by analysing each script line by line. A coding framework was constructed that enabled the extraction of sections of text that related to each code. The codes were readjusted, renamed, collapsed and merged in order to produce a manageable set of basic themes. Each basic theme was named in order to summarise the text sections succinctly and in a way that differentiated it clearly from other basic themes.

The next stage in the analysis involved the construction of thematic networksto identify common groupings of basic themes and group them into organising themes. A title was allocated to each organising theme that reflected the nature of the cluster of basic themes. The organising themes were then grouped into global themes. Each global theme encapsulated the key point of the text. Thematic networks analysis provides a step-by-step process of building theory, starting with basic themes, then merging into organising themes and finally global themes and has common links with other analytical techniques, for example 'Grounded Theory' [35].

For focus group data, the concurrent analysis of field notes, memos and reflections enabled elaboration and refinement of the thematic networks. To enhance the credibility of the data, two researchers, one who had conducted the research and a second who did not collect data but who was experienced in qualitative analysis [33], coded the responses independently. Discussion and consensus was reached related to the systematic development of the three levels of theme.

## Results

### Participants

A total of seven participants, aged 16–19 years, took part in two separate focus groups. Six were primiparous mothers and one was multiparous. Their infants ranged in age from 2 weeks to 6 months and they had breastfed, or were continuing to breastfeed, for between 4 days and 5 months. All were white, reflecting the predominant local ethnic culture.

The demographic characteristics of the midwives and B*f*N supporters are illustrated in Table 2. The age range of the two groups was broadly similar and the majority (n = 19) had worked as a midwife or B*f*N supporter for longer than two years. All participants had attended a breastfeeding training course, most commonly the B*f*N training course (n = 12 B*f*N participants), the 20 hour WHO/UNICEF Breastfeeding Management course (n = 8 midwife participants) and University run breastfeeding courses (n = 3 midwife participants). The B*f*N participants held a variety of additional roles related to working with mothers and infants including, for example, midwifery, heath visiting, and working in other voluntary breastfeeding organisations. Despite this diversity of experience, there was a distinct commonality in approach amongst the B*f*N supporters.

**Table 2:** Participant characteristics

	**Midwives (n = 12)**	**B*f*N^1 ^supporters (n = 12)**
Age range (years)		
20–29	2	1
30–39	5	4
40–49	4	6
50–59	1	1
Years working as a midwife/B*f*N supporter		
< 2	1	4
2 – 5	4	8
> 5	7	0
Other experience working with mothers and infants		
Midwife	12	1
Health visitor	0	1
General practitioner	0	1
Nursery nurse	0	1
NCT^2 ^counsellor	0	5
None	0	3
Breastfeeding training courses attended		
Hospital in-service training	1	0
20-h WHO/UNICEF Breastfeeding		
Management course	8	0
University breastfeeding module/course	3	0
B*f*N training course	0	12

^1^Breastfeeding Network, ^2^National Childbirth Trust

### Themes relating to supportive approaches

Whilst there is wide recognition of the value of the provision of support for breastfeeding mothers, there is often a lack of clarity as to the meaning the term 'support' which makes interpretation of studies problematic [36]. The themes that emerged had striking resonance with the 5-category support schema proposed by Sarafino [37] and was therefore utilised as the framework for discussion of the findings. Sarafino [37](see page 103] refers to there being five components of support:

• Emotional support: "the expression of empathy, caring and concern toward the person";

• Esteem support: '"positive regard for the person, encouragement and agreement with the individual's ideas or feelings";

• Instrumental support: "direct assistance of a practical nature";

• Informational support: "giving advice, directions, suggestions, or feedback about how the person is doing";

• Network support: "provides a feeling of membership in a group of people who share interests and social activities".

Using these components of support as organising themes readily accommodated all the basic themes identified during the analysis. Together these themes were considered to describe a global theme: the provision of holistic support.

### Emotional support

Themes relating to emotional support were expressions of empathy, reassurance and active and focussed listening.

Both midwives and B*f*N supporters placed a great deal of importance on providing reassurance to mothers. Both groups reassured mothers that their experiences were normal "*She would put me out of a job if it wasn't normal to be concerned*"(MW10), "*I would somehow like to convey to her that in the early weeks feeling tired is normal*"(B*f*N11) and that, in fact, many mothers feel the same way they do "*This feeling is expressed by many first time mothers*"(MW6), "*most women find it takes a while to get used to having a baby and caring for her*".

Expressions of empathy were a common feature of the responses of B*f*N supporters in particular, with comments including: "*I would acknowledge that *(it is)* perfectly understandable that she feels the way she does*"(B*f*N10) and "*I might start with an empathetic response – it can feel like a big responsibility being the only one who can feed your baby*"(B*f*N6). Several midwives also offered empathetic understanding "*I would say I understand what she means*"(MW12) and recognised the need to appear approachable and relaxed "*I would sit and talk and show an understanding towards her tiredness*"(MW2).

Related to this was the importance attached by both midwives and B*f*N supporters to creating a caring atmosphere. This included establishing a comfortable environment "*I would expect to be seated and to create a relaxed atmosphere*"(B*f*N4); giving time to the mothers: "*I would allow her time to discuss her worries*"(MW4), "*Give her the chance to discuss her anxieties*"(MW7), "*...making sure I had enough time to listen*"(B*f*N1), "*Let *(her)* talk as long as she wishes*"(B*f*N2); and making their availability explicit. Midwives and B*f*N supporters differed in respect to their accessibility. Availability for midwives involved structured support that fitted within the confines of their roles, with midwives offering "*more frequent visits*" (MW4) and "*contact ...up to 6 weeks for support*" (MW5); whereas B*f*N supporters were able to be more flexible: "*I would offer my home number and encourage *(her)* to use it whenever she needs*" (B*f*N10), "*Offer to visit at home either before or after next clinic visit*" (B*f*N7).

Empathetic understanding requires a process of active and focused listening [38] and this was illustrated in many of the vignette responses, but particularly in those of B*f*N supporters. B*f*N supporters described various components of active listening skills: "*I would ...listen attentively*"(B*f*N4),"*I would listen carefully to her answers try to pick up clues to her specific worries*"(B*f*N7) and "*Give her all my attention, feed back to check I had heard and understood correctly*"(B*f*N2). B*f*N supporters also appeared to be aware of the influence of non-verbal cues "*I would be aware of my own non-verbal communication and body language*"(B*f*N4), "*I would be aware of my body language – being relaxed while she fed*"(B*f*N3).

In general there were fewer responses from the midwives that could be related to the organising theme of emotional support, particularly in relation to the expression of empathy and using active listening skills.

### Esteem support

Both midwives and B*f*N supporters conferred a great deal of importance to enhancing the mothers' feelings of self-worth, ability and being valued both as a mother and in relation to breastfeeding. Central to this theme were attempts to increase confidence "*I would use as many opportunities as appropriate to boost her confidence*"(B*f*N1), "*I would ...give positive comments to boost confidence*"(MW8). Giving praise for choosing to breastfeed ("*I would tell her how special she was to have committed herself to breastfeeding*", B*f*N12), persevering with breastfeeding ("*I would compliment on breastfeeding for 4 weeks*", MW11) and for generally being "*a brilliant mum*"(B*f*N9) and "*doing a superb job*"(MW9) seemed the most commonly used strategy for boosting confidence.

Both groups were keen to avoid undermining confidence by making any criticism of the mothers' management of her breastfeeding and by reinforcing the positive aspects of the situation. For example, in response to vignette 4, where the mothers' partner gives the baby an infant formula feed, B*f*N supporters emphasised that "*your baby will benefit from breast milk at other feeds*"(B*f*N2) and"*You seem to have found a situation that you are all happy with – you will be passing on immunity...and your partner can help when you're tired*" (B*f*N9). Midwives took a practical approach to the situation "*Giving an occasional bottle will not do any harm – better this way than struggling on...and then giving up breastfeeding altogether*"(MW11). Both midwives and B*f*N supporters generally placed great value on the support provided by the partner ("*How fortunate to have such a supportive husband and daddy*", MW10 and "*It can be so nice to see *(partner)* getting involved*", B*f*N6) and positive efforts were made to involve him in discussions about the baby's care ("*Try and see her with her partner and help them devise a way they can support each other and continue breastfeeding*", MW5). Both midwives and B*f*N supporters also emphasised that they would support the mother with whatever decision she made, avoiding judgment and endorsing her decision: "*I would support her in whatever her wishes are*"(B*f*N3); "*Support mum in whatever decision she makes and not be judgemental if she decides to bottle feed*"(B*f*N2); "*I would emphasise it is her choice and I would support whichever she chose*"(MW6).

Respondents, particularly B*f*N supporters, felt it important to 'empower' the women. There was a sense that through empowerment, women could be facilitated to come to their own solutions ("*Quite often women find resources within themselves when they're given the opportunity to talk through a situation. This is a very empowering process*", B*f*N3), be assertive ("*I would try to empower *(her) *to be able to answer in an assertive manner*", B*f*N3), and believe in herself and her abilities,"*My aim would be to empower her – make her believe in herself and be proud of herself for doing the best she can for her baby*", MW6; "*Hopefully empower her to feel a little more confident*", B*f*N6).

Members of both groups emphasised the need to take account of the mothers' young age and to avoid patronising: "*...treat her as an adult. I imagine the slightest whiff of being talked down to would make her withdraw immediately*"(B*f*N6) and "*be careful not to patronise*"(MW9).

### Instrumental support

The most commonly cited source of 'instrumental' support related to the observation of a breastfeed. Both midwives and B*f*N supporters stressed that they would "*check positioning and attachment*"(B*f*N 2) and "*observe baby latching on and feeding*"(MW4). Several midwives suggested using this opportunity to offer reassurance: "*I would fully observe a feed with *(her)*permission and reassure that the positioning is good*"(MW5);"*Observe feed pointing out how well the baby suckles and swallows*"(MW3). Midwives also recognised the desirability of a 'hands off' approach, saying they would let the mother "*place baby to breast herself*"(MW1) and observe the feed "*without putting a hand on *(her)* breast*"(MW5).

Both groups showed a willingness to refer mothers for additional advice – B*f*N supporters referred to health professionals, whereas midwives referred to either other health professionals (a specialist midwife or health visitor) or a "*breastfeeding counsellor*"(MW9).

Several midwives indicated that they would offer to weigh the baby, attaching importance to the baby's weight gain in reassuring the mother that all is well, "*Check her weight gain*"(MW4); "*Weigh baby weekly to reassure*"(MW3). B*f*N supporters on the other hand appeared to be more wary of weight charts: "*I would mention that plotted weights on centile charts are only a rough indicator*"(B*f*N5); "*Weight gain is just one indicator*"(B*f*N10).

Other forms of instrumental support included practical assistance with travel "*I would check whether she could get to the *(Breastfeeding Support)* Centre easily, if not I would offer to bring her*"(B*f*N1) and advocacy "*I would...offer to discuss this with the person concerned*"(MW2); "*Maybe offer to talk to family members...if they are not familiar with breastfeeding*"(B*f*N5).

### Informational support

Informational support was a strong theme in both the midwives' and B*f*N supporters' responses. The ways in which it was offered however, differed between the two groups. B*f*N supporters recognised the need to first assess the mothers' existing knowledge: "*I would find out from her what she already knows. This would help to identify gaps in her knowledge*" (B*f*N8); would seek information about the mothers' prior experiences"*I would try and get an overall picture of how breastfeeding had been going up to now*"(B*f*N11), "*I would ask how she had been getting on, how her breastfeeding experience was going*"(B*f*N1); and would check for understanding "*...checking her understanding along the way*"(B*f*N10). B*f*N supporters were more likely to use open questions as a strategy for encouraging mothers to share their feelings and to assess what information to provide "*Ask her open questions about why she feels the way she does*"(B*f*N2) and "*using open-ended questions to find out whether breastfeeding was pain free....*"(B*f*N1*)*. In contrast, closed questions were a noticeable feature of the midwives' responses, e.g. "*ask if she was upset about the comment*"(MW5), "*I would ask if she felt this way since her discharge from hospital*"(MW4).

B*f*N supporters appeared to be more flexible in their information giving "*I may talk about baby led feeding*"(B*f*N11), "*Possibly tell her...*"(B*f*N3), "*If she was interested, I might explain...*"(B*f*N6), whereas midwives were generally more directive "*I would discuss feeding pattern*"(MW11). B*f*N supporters were also more likely than midwives to explore options and discuss strategies with mothers: "*I would offer options that other mums have tried*"(B*f*N2): "*Explore ways of coping*"(B*f*N6).

The topics discussed by both groups were broadly similar. Both groups said that they would emphasise the benefits of breastfeeding: "*I would...re-emphasise the benefits of breastfeeding*"(B*f*N12); "*Outline benefits of breastfeeding for mum/baby*"(MW10). B*f*N supporters, however, tended to describe these benefits in more specific terms than the midwives "*I would probably explore with her the health benefits of exclusive breastfeeding tactfully, e.g. stimulation of supply, gut flora change...*"(B*f*N2), giving examples of improved health outcome such as "*passing on immunity*"(B*f*N9) or exploring the possible disadvantages of introducing formula by "*making gut and ear infections more likely*"(B*f*N1). The B*f*N supporters also offered information relating to the time taken to establish breastfeeding as a means of providing reassurance and boosting confidence "*...breastfeeding is a skill, just as sending text messages is a skill, and that skills need to be practised and developed*"(B*f*N6); "*I may mention that it can often take 6–8 weeks to get her breastfeeding properly established...*"(B*f*N1).

Both groups provided information to enable the mother to feel reassured that breastfeeding was going well. Information included: monitoring the frequency of wet and dirty nappies "...*ask her *(if the baby has) *plenty of wet and dirty nappies...*"(B*f*N11), "*Use simple reliable signs of success i.e. wet nappies are there any?*"(MW10); ensuring optimal positioning and attachment "*explain the relevance and significance of effective positioning and attachment*"(B*f*N4) and "*Give her certain signs to look for to clarify correct positioning and attachment*"(MW8); and checking that the baby is "*alert and content*"(B*f*N3) and that *she *"*settles for a short while following feeds*"(MW1). B*f*N supporters, however, placed more emphasis on responding to the baby's cues "*I might explain a bit about her baby's cues – about crying being a late sign of hunger*"(B*f*N1), "*I may talk to her about baby led feeding*"(B*f*N11).

B*f*N supporters acknowledged the dangers of giving too much information: "*I would be careful not to offer too much info at one go*"(B*f*N8); "*It is far too easy to talk for long periods and overload someone who is already anxious and stressed with too much detail*"(B*f*N10), whereas midwives appeared more aware of the problems of conflicting advice, commenting that the "*Same midwife *(should)* visit frequently so conflicting advice is not given*"(MW1). B*f*N supporters were also far more likely to offer other forms of information, in particular the loan of books; only one midwife (MW5) mentioned an additional information source – the loan of a video "*to show correct positioning*".

### Network support

The importance of having access to network support, particularly from family and friends, was acknowledged by both midwives and B*f*N supporters, although this theme was stronger in the B*f*N responses. Only the B*f*N supporters explored the existing network support the mother had access to "*It would be useful to ascertain what support she has at home*"(B*f*N8) and indeed "*what *(support) *would she like?*"(B*f*N2). Midwives tended to focus on the ways in which the mothers' partner could provide support and described a range of practical ways in which fathers could help: "*maybe he could bath *(the baby)* and get him ready for bed before *(mum)* gave him his feed*"(MW9), "*Discuss expression of breastmilk which her partner could give if he wanted to play a part*"(MW11). B*f*N supporters discussed the role of the father "*I would discuss with her ways to involve and validate her partner*"(B*f*N8), her family "*Possibly finding support from her family members during the day when her partner was at work*"(B*f*N8), and peers "*Take along someone supportive (baby's father, her mother or a friend)*"(B*f*N6) in supporting the breastfeeding mother. B*f*N supporters also acknowledged that sometimes, such sources of support may be undermining "*Some dads do feel that they are missing out if mum is breastfeeding*"(B*f*N9), "*If *(the mother)* is living with her parents her mother's feeding choices may be being imposed on *(the baby)"(B*f*N6). Although BfN supporters were more likely to recommend that the mother access local support groups as a source of "*embodied role models*" (B*f*N5), midwives also acknowledged the importance of such groups "*to enable *(the mother) *to be reassured by other people in her situation*"(MW11).

As well as facilitating access to one-to-one network support, B*f*N supporters also provided information about other support media available to mothers, such as the telephone "*Giving her the Supporterline number and times it was open*"(B*f*N1) and the internet "*I might point out a *'*good*'* ... breastfeeding website where she could... *'*chat*'* to others*"(B*f*N6).

## Discussion

This study explored the similarities and differences in the approaches of midwives and qualified breastfeeding supporters (B*f*N supporters) in supporting adolescents who were breastfeeding their babies. The use of vignettes provided a way in which participants could spend time to reflect upon and provide a considered response to the four scenarios. This enabled them to describe how they would respond to each situation in an ideal scenario. Some of the constraints upon respondents in a practice setting were evident in responses, such as the shorter period for which midwives might be able to visit within the confines of their roles. However, the full constraints upon either group could not be elicited in this context. Thus, in situations where time pressures may be felt the actions of respondents might be different [13]. Equally, in situations in which midwives had competing agendas related to the 'needs' of the institution rather than the mother, they may behave differently [39,40]. The degree to which the ideal response may differ from actual practice could only be elicited through ethnographic studies. Ethnographies of postnatal ward settings have been conducted [13,41,42] but not within B*f*N centres. A comparative ethnography of the two settings may reveal larger differences between groups.

The samples of midwives and B*f*N supporters were small and incorporated an element of self-selection, thus limiting generalisability. Despite the random selection of 50 midwives, only twelve agreed to participate. The B*f*N supporters were likely to be more representative of their overall group due to the small numbers practising in this capacity, however, as randomisation did not take place there remains an element of selection bias. It is likely that in both cases a self-selected group will be more committed to breastfeeding and may be more knowledgeable. This is more likely to be the case for midwives, as it could be argued that all B*f*N supporters must be committed to offer up their time voluntarily to counsel breastfeeding women.

Although these findings relate specifically to midwives and a particular group of breastfeeding supporters working in the UK, it is possible that the approaches described would reveal commonalities with the supportive approaches of similar groups in other contexts. As stated, the B*f*N uses similar training and approaches to international breastfeeding support organisations, such as LLL and ABM. Likewise midwifery training in the UK and the institutional settings within which it is practiced has similarities within other countries that also adopt a biomedical model to birth and breastfeeding. Similarly, it is not unreasonable to suggest that these approaches, although specific to scenarios involving adolescent mothers, would be comparable to those used with older mothers. However, further research is needed in order to elucidate these assumptions.

A further limitation of the study relates to the lack of diversity amongst the focus group participants, which was consequently reflected in the nature of the vignettes. Due to financial and resource constraints, non-English speakers were excluded from the study and, combined with a predominantly white local ethnic culture, resulted in an entirely white Caucasian sample. The focus group sample was further reduced by excluding those whose baby was unwell and/or admitted to the neonatal unit as these situations would create different sets of circumstances that would warrant a separate research project. Thus the vignettes specifically reflected the breastfeeding experiences of white, English speaking mothers of well babies which, in turn, restricts the generalisability of the midwives' and BfN supporters' responses to the vignettes. Future research should consider the breastfeeding experiences of more diverse populations in order to elicit further similarities and differences in the approach of midwives and qualified breastfeeding supporters.

A number of similarities in the supportive approaches of midwives and B*f*N supporters were identified. Both midwives and B*f*N supporters indicated that they would provide emotional support, placing emphasis upon reassurance and creating a caring atmosphere. With regard to esteem support, both groups stressed the importance of enhancing the mother's feelings of self worth, ability and being valued. They emphasised avoidance of language that could undermine confidence. Instrumental support was highlighted by both midwives and B*f*N supporters, particularly with regards to the importance of teaching and checking positioning and attachment. Informational support was a strong theme in both the midwives' and B*f*N supporters' responses, with the topics discussed revealing broad similarities. Both groups emphasised the value of accessing network support from family, peer groups and voluntary groups such as the B*f*N.

There were numerous differences between the supportive approaches of midwives and B*f*N supporters. With regard to emotional and esteem support, B*f*N supporters placed considerably more emphasis upon empathetic understanding, the use of active listening skills and on empowering women. This almost certainly reflects the specific emphasis upon these as aspects of person-centred counselling in their curriculum. With regard to instrumental support, midwives, more than B*f*N supporters, referred to a 'hands-off' approach, although only ethnographic work would verify the extent to which this was practiced in either group. Midwives appeared to see offering to weigh the baby as a key way of supporting, whereas B*f*N supporters were quite wary of weighing. The wariness on the part of the B*f*N supporters to weighing may reflect a more in depth knowledge of the challenges that weighing may create to breastfeeding women, as recently summarised by Sachs et al. [43,44]. B*f*N supporters also placed more emphasis upon responding to baby cues rather than using more instrumental approaches such as the measurement of external parameters to ascertain well-being and growth. This may reflect a more extensive education upon this aspect of their role than in that of midwives.

Although both groups placed a large emphasis upon informational support, B*f*N supporters highlighted the need to elicit the mothers' existing knowledge, checking understanding through use of open questions and utilised more tentative language with regard to information giving. The midwives came across as more directive and gave more examples of closed questions. This is perhaps the largest difference between the two groups. It may reflect, in the case of midwives, the bureaucratic and institutional constraints upon them making it difficult, if not impossible, to take time and touch base with women [13,39,40,45]. It would be interesting to see how B*f*N supporters operated in a situation where they had several women waiting for their attention.

The B*f*N supporters appeared to refer to the long term nature of breastfeeding and this may reflect their anticipated ongoing, rather than short term, involvement with women. This resonates with the findings of Cloherty et al [41] who note that midwives have a tendency to offer short-term solutions without a full understanding of the long-term implications. This reflects their short-term involvement with women, particularly if based primarily in a hospital setting. Both groups emphasised the importance of accessing network support both from family, peer groups and support networks such as the B*f*N.

The greater emphasis upon emotional and esteem support of B*f*N supporters almost certainly reflects the considerable emphasis upon person-centred approached within the B*f*N curriculum. It could be argued that the provision of emotional and esteem support is the main focus of the person-centred approach. Certainly the presence of the core conditions of unconditional positive regard, empathetic understanding and congruence within the mother/supporter relationship could be expected to raise self-esteem and self-belief and thus empower the mother to develop her own coping strategies. Mearns and Thorne [26] describe the central tenet of the person-centred approach as a belief in abdicating power in order to empower. This is clearly illustrated both in the language used by the B*f*N supporters in their vignette responses and in their descriptions of the other communication skills, particularly active listening, that they would use in their attempts to build supportive relationships. The abdication of power in a relationship is arguably more difficult for a midwife who has a professional responsibility not only for the well-being of the mother but also for that of her baby. This may well account for the more directive approach to support that appears to have been demonstrated by the participating midwives. It should also be recognised that a greater focus on informational or instrumental support may be preferred by some mothers; Natiello [46] described the frustration of some recipients of the person-centred approach when expected practical solutions or advice were not forthcoming. Indeed, Mearns and Thorne [26] acknowledge that person-centred counselling is only one form of helping and that it is not a template for all helping relationships let alone for effective human relating in general.

It would therefore seem inappropriate to expect that a uniform approach to support should be adopted by these two very different groups. Not only would it not be feasible but it would also fail to provide for the individual preferences of mothers. Perhaps the greater concern is that the support, suggestions, strategies or information offered by both groups should have a common evidence base, thus avoiding the common complaint from breastfeeding women of inconsistent or conflicting advice. With perhaps the exception of the issue of weighing, this study illustrated that both groups were drawing on the same evidence base in their attempts to offer support even if their approaches were somewhat different.

## Conclusion

Overall these findings reflect the differences in the curricula and subsequent approaches of B*f*N supporters and midwives. As stated, follow up ethnographic work is needed to assess these differences out in the practice areas. Policy and practice recommendations cannot be made without supplementary observational work and research that focuses specifically upon how the different approaches are received and experienced by parents. Such research may of course reveal that these differences are valued by women, rather than identifying a 'right' way to offer support. Given the recommendations, referred to above, to offer some form of supplementary support to breastfeeding women, it would appear to be a pragmatic option to consider the employment of qualified breastfeeding supporters within health care systems. This would, however, require ongoing evaluation as with any new initiative, as was recently conducted in the UK [10].

## Competing interests

The author(s) declare that they have no competing interests.

## Authors' contributions

VHM & FD were involved in the conception, design and coordination of the study, in the analysis and interpretation of the data, and in the writing of the manuscript. SB was employed as a research assistant on this project and participated in the collection, analysis and interpretation of the data. CS was involved in the analysis and interpretation of the data. The research was not conducted in the maternity unit in which SB and CS were employed. All authors read and approved the final manuscript.
